# Prognostic and Predictive Value of BGN in Colon Cancer Outcomes and Response to Immunotherapy

**DOI:** 10.3389/fonc.2021.761030

**Published:** 2022-01-11

**Authors:** Zi-Xuan He, Sheng-Bing Zhao, Xue Fang, Ji-Fu E, Hong-Yu Fu, Yi-Hang Song, Jia-Yi Wu, Peng Pan, Lun Gu, Tian Xia, Yi-Long Liu, Zhao-Shen Li, Shu-Ling Wang, Yu Bai

**Affiliations:** ^1^Department of Gastroenterology, Changhai Hospital, Second Military Medical University/Naval Medical University, Shanghai, China; ^2^Department of Colorectal Surgery, Changhai Hospital, Second Military Medical University/Naval Medical University, Shanghai, China; ^3^College of Basic Medicine Sciences, Second Military Medical University/Naval Medical University, Shanghai, China

**Keywords:** BGN, colon cancer, tumor microenvironment, immunosuppression, immunotherapy

## Abstract

**Background:**

Colon cancer is one of the most frequent malignancies and causes high mortality worldwide. Exploring the tumor-immune interactions in the tumor microenvironment and identifying new prognostic and therapeutic biomarkers will assist in decoding the novel mechanism of tumor immunotherapy. BGN is a typical extracellular matrix protein that was previously validated as a signaling molecule regulating multiple processes of tumorigenesis. However, its role in tumor immunity requires further investigation.

**Methods:**

The differentially expressed genes in three GEO datasets were analyzed, and BGN was identified as the target gene by intersection analysis of PPIs. The relevance between clinical outcomes and BGN expression levels was evaluated using data from the GEO database, TCGA and tissue microarray of colon cancer samples. Univariable and multivariable Cox regression models were conducted for identifying the risk factors correlated with clinical prognosis of colon cancer patients. Next, the association between BGN expression levels and the infiltration of immune cells as well as the process of the immune response was analyzed. Finally, we predicted the immunotherapeutic response rates in the subgroups of low and high BGN expression by TIS score, ImmuCellAI and TIDE algorithms.

**Results:**

BGN expression demonstrated a statistically significant upregulation in colon cancer tissues than in normal tissues. Elevated BGN was associated with shorter overall survival as well as unfavorable clinicopathological features, including tumor size, serosa invasion and length of hospitalization. Mechanistically, pathway enrichment and functional analysis demonstrated that BGN was positively correlated with immune and stromal scores in the TME and primarily involved in the regulation of immune response. Further investigation revealed that BGN was strongly expressed in the immunosuppressive phenotype and tightly associated with the infiltration of multiple immune cells in colon cancer, especially M2 macrophages and induced Tregs. Finally, we demonstrated that high BGN expression presented a better immunotherapeutic response in colon cancer patients.

**Conclusion:**

BGN is an encouraging predictor of diagnosis, prognosis and immunotherapeutic response in patients with colon cancer. Assessment of BGN expression represents a novel approach with great promise for identifying patients who may potentially benefit from immunotherapy.

## Introduction

Global Cancer Statistics of 2020 demonstrates that colorectal cancer (CRC) ranks 3^rd^ in terms of the most frequent malignancy and 2^nd^ in terms of tumor-related death. CRC accounts for 9.8% of the total cancer incidence with nearly 1.9 million new cases and 9.2% of the total case mortality with 935,000 deaths annually (1, 2). Although diagnosis of CRC has improved due to early detection through colonoscopy screening and advances in imaging techniques such as CT colonography or PET-CT, approximately 25% of patients still experience advanced diseases (3), which only achieve modest benefits from conventional therapeutic strategies, including surgery, chemotherapy, and radiotherapy (4–6). Immunotherapy holds promise in cancer treatment, providing a novel treatment tool for patients with advanced or drug-resistant colorectal cancer (7). However, responses to immunotherapy vary significantly among different types of colorectal cancer patients (8, 9). Therefore, predicting the efficacy of immunotherapy and finding efficacy-related biological markers are of particular importance for the treatment of colorectal cancer (10, 11).

Recent advances have emphasized the significance of the tumor microenvironment (TME), accounting for approximately 90% of the gross tumor volume. In fact, nontumoral cells including immune cells, cancer-associated fibroblasts (CAFs) or extracellular matrix (ECM) exist in the great majority of solid tumors (12, 13). Within this intratumor microenvironment, both tumor cells and stroma contribute to non-cellular components, such as extracellular matrix, which are largely characterized and associate with tumor invasiveness and metastatic abilities (14, 15). Exploring the impact of cellular composition in the TME would assist in decoding the regulation of the microenvironment by tumors.

BGN is a classic type of extracellular matrix protein that is essential role in mediating the morphology, growth, differentiation and migration of epithelial cells (16). The function of the BGN depends on its microenvironmental context as a structural or signaling molecule. Initially, the function of BGN was mainly to maintain the structural integrity of the ECM (17). In recent years, however, BGN has been regarded as a signaling molecule mediating various steps of tumorigenesis within recent years (16). Aberrant expression of BGN in tumors indicate its promoting effects in migration and invasion abilities of tumor cells (18). Previous studies have found that the up-regulation of BGN in a variety of solid tumors (19–21) and its potential diagnostic and prognostic value in ovarian cancer (21), prostate cancer (22), gastric cancer (23) and colorectal cancer (24, 25). However, a paucity of studies have systematically assessed the function of BGN in tumor immunity. In our current work, which combines data from TCGA and GEO public databases as well as tissue microarrays, we validated the clinical implication of BGN expression in colon cancer samples and utilized multiple bioinformatics approaches to investigate the underlying immunosuppressive mechanisms of BGN in the TME as well as its potential role in predicting immune checkpoint blocker (ICB) immunotherapy responses in colon cancer patients.

## Results

### Identification of DEGs

**Figure 1:**
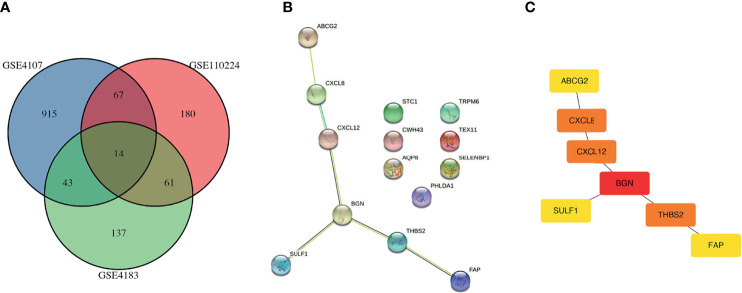
Identification of overlapping DEGs. **(A)** Venn plots of overlapping DEGs in GSE4107 dataset, GSE110224 dataset and GSE4183 dataset. **(B)** PPI of 14 DEGs using the STRING database (https://string-db.org). **(C)** PPI network of DEGs were constructed and visualized by Cytoscape software. PPI, protein-protein interaction; DEG, differentially expressed gene.

First, three GEO datasets including 44 colon cancer tissues and 35 normal tissues were enrolled to identify differential genes. According to the cutoff criteria, 1039, 396, and 256 differentially expressed genes were extracted from GSE4107, GSE110224, and GSE4183 by the GEO2R online tool, respectively. As shown in **Figure 1A**, 14 differentially expressed genes with overlapping expression were screened. Subsequently, a protein-protein interaction network was constructed by the STRING tool (**Figure 1B**) and then uploaded the common PPI network into Cytoscape software. Based on the degree scores generated by the CytoHubba module, seven genes, ABCG2, CXCL8, CXCL12, BGN, SULF1, THBS2, and FAP, were identified as potential hub genes (**Figure 1C**). Among them, BGN had the highest degree score and was identified as the target gene for subsequent functional analysis and validation.

### Assessment of BGN Expression in Colon Cancer and Normal Tissues

**Figure 2:**
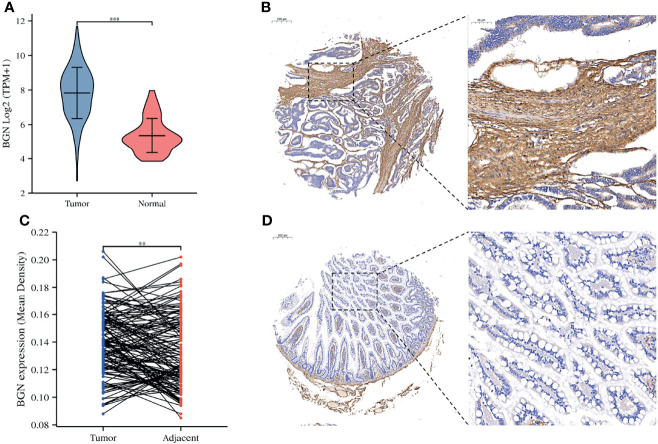
The expression levels of BGN were significantly upregulated in patients with colon cancer. **(A)** The mRNA expression of BGN in the normal and tumor samples in TCGA. Data are represented as the mean ± SD. The Y-axis represents log2 (TPM+1) transformed RNA seq expression data. Wilcoxon rank sum test served as the statistical significance test. **(B, D)** Immunohistochemistry staining of example colon cancer patients with positive BGN staining in tumor tissues and the negative BGN staining in adjacent tissues. **(C)** Paired differentiation analysis for mean density of BGNs detected by immunohistochemistry staining sin tumor and paired adjacent tissues deriving from tumor microarray. The Wilcoxon signed rank test was used for comparison. Significant differences referred ass: **p < 0.01, ***p < 0.001.

We compared the mRNA expression levels of BGN among 435 colon cancer samples and 41 normal samples in TCGA. In patients with colon cancer, the transcriptional levels of BGN were markedly upregulated (**Figure 2A**). To investigate the protein expression patterns of BGN, an IHC assay was performed in tumor microarray (TMA) sections. BGN showed an extracellular matrix staining pattern (**Figures 2B, D**). The expression levels of BGN protein were significantly elevated in tumor tissues, as compared with in the adjacent noncancerous tissues (MOD = 0.140 ± 0.023 *vs* 0.133 ± 0.026, p = 0.003) (**Figure 2C**). We then analyzed the association between the upregulation of BGN and clinicopathological variables. As shown in **Table 1**, a total of 84 patients (58.3%) stained positive for BGN expression in tumor tissues. Upregulation of BGN was markedly correlated with tumor size (p = 0.023), serosa invasion (p = 0.002) and surprisingly, length of hospitalization (p = 0.038). These findings above indicate that upregulation of BGN might be a potential biomarker for assessing the degree of malignancy in colon cancer.

**Table 1:** Association between BGN expression and clinicopathological factors of colon cancer patients.

Variables	Number of cases	Up-regulation of Biglycan numbers (%)		p value
		Positive (N=84)	Negative (N=60)	
**Gender**				
Male	99	58(69.05%)	41(68.33%)	0.927
Female	45	26(30.95%)	19(31.67%)	
**Age (years)**				
>=60	89	53(63.10%)	36(60.00%)	0.706
<60	55	31(36.90%)	24(40.00%)	
**Tumor location**				
Colon	83	52(61.90%)	31(51.67%)	0.220
Rectum	61	32(38.10%)	29(48.33%)	
**Tumor size**				
T1/T2	28	11(13.10%)	17(28.33%)	**0.023**
T3/T4	116	73(86.90%)	43(71.67%)	
**Lymph node metastasis**				
N0	68	41(48.81%)	27(45.00%)	0.652
N1/N2	76	43(51.19%)	33(55.00%)	
**Distant metastasis**				
M0	112	62(73.81%)	50(83.33%)	0.175
M1	32	22(26.19%)	10(16.67%)	
**length of hospitalization**				
< 10 days	44	20(23.81%)	24(40.00%)	**0.038**
<=10 days	100	64(76.19%)	36(60.00%)	
**Tumor type**				
ulcerative	33	18(21.43%)	15(25.00%)	0.615
Non-ulcerative	111	66(78.57%)	45(75.00%)	
**Tumor differentiation**				
Well to moderate	128	76(92.68%)	52(89.66%)	0.528
Poor	12	6(7.32%)	6(10.34%)	
**Tumor stage**				
I	19	8(9.52%)	11(18.33%)	0.124
II/III/IV	125	76(90.48%)	49(81.67%)	
**Disease duration**				
>3 months	45	28(33.73%)	17(29.31%)	0.580
<=3 months	96	55(66.27%)	41(70.69%)	
**Serosa invasion**				
Yes	115	73(86.90%)	42(70.00%)	**0.002**
No	29	11(13.10%)	18(30.00%)	

Length of stay was defined as the number of days the patient was hospitalized for surgical treatment of colon cancer. One day was defined as a hospital stay crossing midnight and was determined by independent physician review from medical records and patient reports. The p value was calculated by the Chi-square test. Bold numbers indicate statistically significant values (p < 0.05).

### Prognostic Value of BGN in Colon Cancer Patients

**Figure 3:**
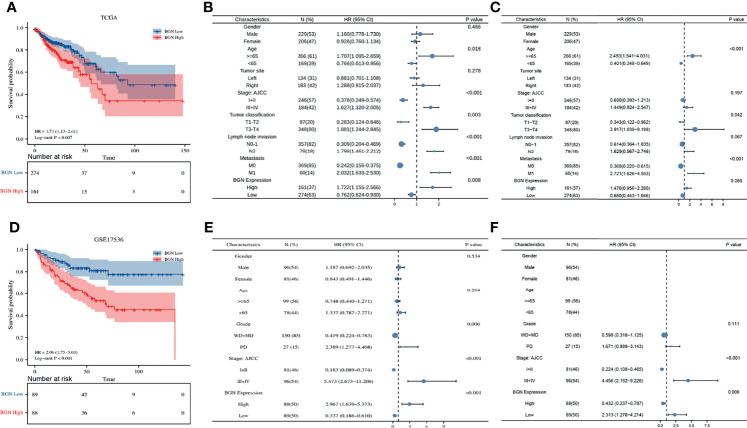
The expression levels of BGN are correlated with overall survival and clinicopathological characteristics of colon cancer patients in TCGA and GSE17536 dataset. **(A, D)** Kaplan-Meier analysis of overall survival in colon cancer patients with different BGN expression. The significance was calculated by the Log-rank test. Number of patients remaining in follow-up at each time point is reported in the box below the figure for each group. **(B, C)** Univariate and multivariate cox regression analysis of clinicopathological characteristics and BGN expression with overall survival in TCGA. **(E, F)** Univariate and multivariate cox regression analysis of clinicopathological characteristics and BGN expression with overall survival in GSE17536. p < 0.05 was considered statistically significant. Whiskers represent the 95% confidence interval of the HR value, and dots represents the values of HR.

We next explored the association between BGN expression levels and outcomes of colon cancer patients in TCGA and GEO database. Our results manifested that higher BGN expression was linked to poorer overall survival (OS) in patients with colon cancer in TCGA (p = 0.007) and GSE17536 dataset (p < 0.001) (**Figures 3A, D**). Additionally, univariable and multivariable Cox regression analyses were conducted for identifying the relevant clinicopathological factors of colon cancer patients in TCGA and GSE17536, respectively. As a result, BGN was determined to be an independent prognostic biomarker that could be applied to predict poor OS in GSE17536 (p = 0.006) (**Figures 3E, F**). In the TCGA database, the prognostic significance of BGN as an independent prognostic factor was not statistically significant (p = 0.080) (**Figures 3B, C**).

### The Correlation Between BGN Expression Levels and the TME Immune and Stromal Scores

**Figure 4:**
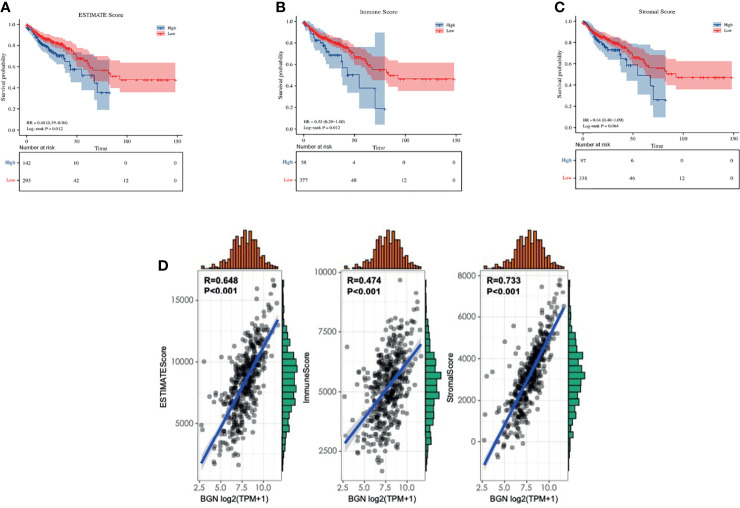
The correlation between BGN expression and tumor microenvironment scores in colon cancer. **(A–C)** ESTIMATE score, immune score and stromal score in predicting overall survival of colon cancer based on Kaplan-Meier analysis. The significance was calculated by the Log-rank test. **(D)** BGN expression is strongly associated with estimate score, immune score and stromal score in colon cancer based on Pearson correlation analysis. p < 0.05 was considered statistically significant. The above analysis were based on TCGA expression data.

Interactions of the TME and tumor cells are of great importance for tumor progression and the effect of immunotherapy. Based on the transcriptome data of TCGA, immune and stromal proportions of colon cancer tissues were calculated by a well-established ESTIMATE algorithm. Higher immune or stromal score represent a greater proportion of immune or stromal components in the TME, while ESTIMATE score represent the combined proportion of them. Through Kaplan–Meier analysis, we found that higher ESTIMATE score and immune score were both associated with poor OS (both p = 0.012) in colon cancer patients, except for the stromal score (p = 0.064) (**Figures 4A**–**C**), which is consistent with previously reported results (26). Moreover, correlation analysis demonstrated that BGN expression correlated with the immune score, stromal score and ESTIMATE score (Pearson r = 0.474, 0.733 and 0.648, respectively, all p < 0.001) in colon cancer (**Figure 4D**). Further analysis suggested that this association presented in a stage-independent mode (**Supplementary Figure 1**). These results suggested that the independent prognostic role of BGN in colon cancer might be associated with alterations in the TME.

### The Role of BGN in Modulating Macrophage Polarization in Colon Cancer

**Figure 5:**
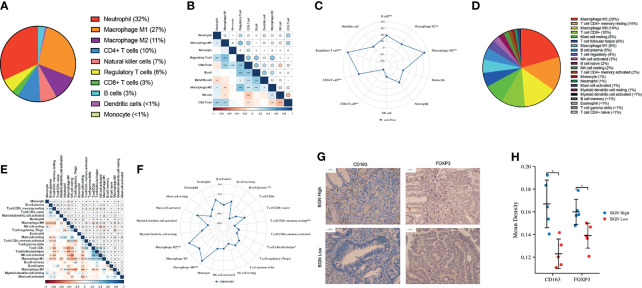
TIIC profile in colon cancer samples and correlation analysis. **(A, D)** Pieplots showing the estimated proportion of different kinds of TIICs in colon tumor samples prediceted by **(A)** quanTIseq and **(D)** CIBERSORT. **(B, E)** Pearson correlation matrix of the different TIIC proportions in the colon cancer microenvironment quantified by **(B)** quanTIseq and **(E)** CIBERSORT. The size of each bubble and shadow of each tiny color box both represented a corresponding correlation value between two cells. Asterisks in each tiny box, indicating the p-value of the correlation between two cells. **(C, F)** Radar chart showing the correlation between BGN expression and different proportions of TIIC analyzed by **(C)** quanTIseq and **(F)** CIBERSORT. **(G)** Representative images of IHC staining for CD163 and FOXP3 in BGN high and low expression groups. **(H)** Quantification of IHC staining intensity for CD163 and FOXP3 in BGN high and low expression groups. Wilcoxon rank sum test served as the statistical significance test. *p < 0.05, **p < 0.01. ***p < 0.001.

For further investigation of the relationship between BGN and the subtypes of immune cell in colon cancer tissues, the proportion of tumor-infiltrating immune cells (TIICs) was segmented by using quanTIseq in TCGA. Among these TIIC subtypes, macrophages (M1 + M2) accounted for approximately 38%, neutrophils accounted for approximately 32%, CD4+ T cells accounted for 10% and NK cells accounted for 7% in colon cancer tissues (**Figure 5A**). The association between BGN expression levels and immune cell infiltration in colon cancer was explored (**Figure 5B**). Pearson correlation analysis indicated that BGN expression exhibited the most significantly related to the macrophage M2 population and regulatory T cell (Tregs) population (r = 0.34, 0.36, respectively, both p < 0.001) (**Figure 5C**). Next, we utilized the CIBERSORT method to validate the association of BGN expression and immune components by constructing 22 types of immune cell profiles in colon cancer and analyzed the proportion of TIICs (**Figures 5D, E**). The top three largest fractions of immune cells were macrophage M2 (20%), CD4+ memory resting T cell (14%) and macrophage M0 (14%) (**Figure 5D**), which revealed that macrophages may play a potential role in tumor immunity. Subsequent correlation analysis revealed that a total of five types of TIICs were strongly associated with BGN expression. Two of the TIICs (M0 macrophages and M2 macrophages) were in positive correlation with BGN expression. Three TIICs were correlated with BGN expression negatively, comprising plasma B cells, follicular helper T cells and resting memory CD4+ T cells (**Figure 5F**). Moreover, association between BGN expression and cell surface markers of diverse types of TIICs were assessed. Pearson correlation coefficients were calculated, and the results indicated that BGN presented the strongest correlations with TIIC markers for monocytes (CD86, CD115), tumor-associated macrophages (TAMs) (IL-10, CCL2, CD68), M2 macrophages (CD163, VSIG4, and MS4A4A) and Tregs (FOXP3, CCR8, TGFβ) (**Table 2**). In addition, the protein expression levels of M2 macrophage marker (CD163) and Tregs marker (FOXP3) were further validated in patient-derived tissue samples. Colon cancer samples with high BGN expression (n = 5) and low BGN expression (n = 5) were used for IHC analyses. As expected, in comparison with low BGN expression samples, both CD163 and FOXP3 were significantly upregulated in high BGN expression samples (**Figures 5G, H**). The above results demonstrated that high BGN expression might facilitate the polarization of M2 although further efforts are required to verify the underlying mechanisms.

**Table 2:** Correlation analysis between BGN and related surface markers of immune cells.

Description	Gene markers	Cor	P
CD8+T cell	CD8A	0.307	***
	CD8B	0.177	***
T cell(general)	CD3D	0.277	***
	CD3E	0.384	***
	CD2	0.340	***
B cell	CD19	0.243	***
	CD79A	0.338	***
Monocyte	CD86	0.614	***
	CD115(CSF1R)	0.642	***
TAM	CCL2	0.632	***
	CD68	0.531	***
	IL10	0.434	***
M1 Macrophage	INOS(NOS2)	-0.153	**
	IRF5	0.323	***
	COX2(PTGS2)	0.198	***
M2 Macrophage	CD163	0.646	***
	VSIG4	0.628	***
	MS4A4A	0.582	***
Neutrophils	CD66b(CEACAM8)	-0.198	***
	CD11b(ITGAM)	0.703	***
	CCR7	0.377	***
Natural killer cell	KIR2DL1	0.180	***
	KIR2DL3	0.157	***
	KIR2DL4	0.157	***
	KIR3DL1	0.244	***
	KIR3DL2	0.223	***
	KIR3DL3	0.020	0.6649
	KIR2DS4	0.158	***
Dendritic cell	HLA-DPB1	0.544	***
	HLA-DQB1	0.339	***
	HLA-DRA	0.451	***
	HLA-DPA1	0.496	***
	BCDA-1(CD1C)	0.353	***
	BCDA-4(NRP1)	0.765	***
	CD11c(ITGAX)	0.711	***
Th1	T-bet(TBX21)	0.388	***
	STAT4	0.291	***
	STAT1	0.388	***
	IFN-γ(IFNG)	0.185	***
	TNF-α(TNF)	0.309	***
Th2	GATA3	0.488	***
	STAT6	0.062	0.1874
	STAT5A	0.311	***
	IL13	0.270	***
Tfh	BCL6	0.560	***
	IL21	0.221	***
Th17	STAT3	0.304	***
	IL17A	-0.236	***
Treg	FOXP3	0.567	***
	CCR8	0.529	***
	STAT5B	0.316	***
	TGFβ(TGFB1)	0.723	***

The p value was calculated by the Pearson correlation analysis. Significant differences referred as: ***p < 0.001.

### Identification of the Potential Interaction of BGN in Colon Cancer Immune Responses

**Figure 6:**
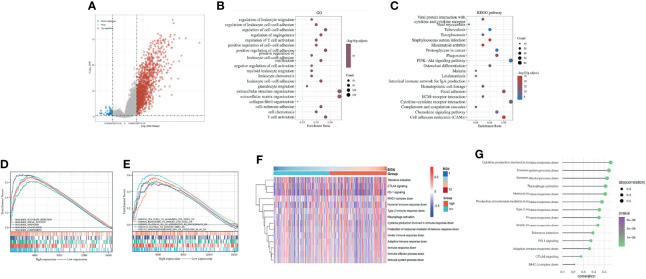
Identification of the correlation between BGN and the regulation of immune responses in colon cancer. **(A)** Volcano plot of the DEGs expression between BGN high and low subgroups. The blue and red dots represented the significantly downregulated and upregulated genes, respectively; The gray dots represented the genes without differential expression. **(B, C)** GO and KEGG results for differential expression genes. The X-axis represents gene ratio and the Y-axis represents different enriched pathways. **(D, E)** The enriched gene sets in HALLMARK and C7 collections by samples of DEGs. Each line representing one particular gene set with unique color. Only gene sets with NOM p-value < 0.05 and FDR q-value < 0.05 were considered significant. **(F)** Heatmap showing BGN-associated GSVA scores of 14 innate and adaptive immunity-related gene sets. **(G)** Pearson correlation between the GSVA scores of 14 innate and adaptive immunity-related gene sets and the expression level of BGN in colon cancer.

We then analyzed the differentially expressed genes (DEGs) between the high and low expression subgroups by the median level of BGN expression. A total of 1483 upregulated genes and 50 downregulated genes were obtained (adj.p-value < 0.05, fold change > 1.5 or < -1.5, **Figure 6A** and **Supplementary Table 1**). In subsequent GO enrichment analysis, DEGs were predominantly concentrated in extracellular organization as well as in immune-related functions, comprising regulation of leukocyte migration and regulation of T cell activation (**Figure 6B** and **Supplementary Table 2**). KEGG analysis results demonstrated that cell adhesion molecules (CAMs), cytokine-cytokine receptor interactions together with the PI3K−Akt signaling pathway were significantly enriched (**Figure 6C** and **Supplementary Table 3**). Moreover, a gene set enrichment analysis (GSEA) was implemented. For HALLMARK gene sets defined by MSigDB, the DEGs in BGN high-expression group showed significant enrichment for immunological activities, such as inflammatory response or complement and interferon response (**Figure 6D** and **Supplementary Table 4**). For the C7 collection, which was defined as an immunologic gene set, multiple functional gene sets that were involved in immunosuppression were enriched (**Figure 6E** and **Supplementary Table 4**). These above results implied that BGN may act as a potential index for the immune status of the TME. To examine this further, we clarified the potential impact of BGN in the tumor immune response process. Manually curated gene sets associated with innate or adaptive immune responses were applied to quantify the immune status (**Figure 6F**). As a result, the immune response regulated by a series of immune cells tended to be “suppressive” with increasing of BGN expression, implying that BGN might also be involved in a negative interaction with the immune responses of colon cancer (**Figure 6G**).

### Potential Role of BGN Expression in Predicting Immunotherapy Responses in Colon Cancer

**Figure 7:**
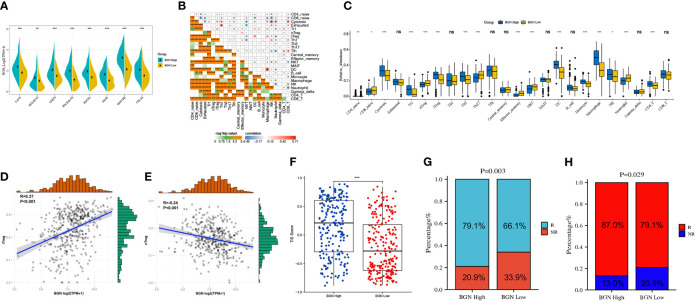
Subgroups divided by levels of BGN expression predict potential immunotherapy responses of colon cancer. **(A)** Immune-checkpoint-relevant genes expressed in high and low BGN subgroups. Wilcoxon rank sum test served as the statistical significance test. **(B)** Pearson correlation matrix of the different TIIC proportions in the colon cancer microenvironment quantified by the ImmunCellAI. **(C)** The fraction of TILCs in BGN high and low subgroups. Wilcoxon rank sum test served as the statistical significance test. **(D, E)** The correlation between the abundance of **(D)** iTreg cells or **(E)** nTreg cells and the expression level of BGN. **(F)** T-cell inflammatory signature (TIS) scores across BGN subgroups. (Wilcoxon rank sum test, p < 0.001) **(G, H)** Response rates to immunotherapy of patients with colon cancer from the TCGA cohort predicted by the **(G)** ImmunCellAI (Chi-square test, p = 0.003) and **(H)** Tumor Immune Dysfunction and Exclusion (TIDE) web program (Chi-square test, p = 0.029) in the high or low BGN subgroups. *p < 0.05; **p < 0.01; ***p < 0.001; ns: p > 0.05 no significance.

Primarily, LAG3, SIGLEC15, CD274, PDCD1LG2, PDCD1, TIGIT, HAVCR2 and CTLA4 were selected as immune checkpoint signature genes (27). Comparing with the BGN low-expression subgroup, all these genes exhibited elevated expression levels in the BGN high-expression subgroup (**Figure 7A**). Subsequently, we quantified the relative abundance of 24 TIICs in the TME using ImmuCellAI. As shown in **Figure 7B**, the correlation heatmap revealed various degrees of correlation between different TIIC subgroups. Notably, the proportion of TIICs varied significantly between the high and low expression subgroups of BGN (**Figure 7C**). Among them, we focused on two subgroups of Treg cells, induced Treg cells (iTregs) and natural Treg cells (nTregs). A positive correlation between BGN expression and the number of iTreg cells was found (**Figure 7D**), while it was the opposite with the number of nTreg cells (**Figure 7E**). To further investigate the potential contribution of BGN in predicting immunotherapy response of colon cancer, T-cell inflammatory signaling (TIS) scores were calculated in both high and low BGN expression subgroups. Patients with high BGN expression presented higher TIS score (p < 0.001), which was previously reported to correlate with the efficacy of anti-PD-1 inhibitor pembrolizumab (28) (**Figure 7F**). Moreover, immunotherapy response rates in patients with colon cancer were predicted by ImmuCellAI and TIDE algorithms. Patients with high BGN levels were more likely to respond to immunotherapy (79.1%) than those with low BGN levels (66.1%), as predicted by ImmuCellAI (**Figure 7G**). The TIDE algorithm similarly reached the conclusion that the immunotherapy response rates in patients with high and low BGN expression subgroups were 87% and 79.1%, respectively (**Figure 7H**). Collectively, BGN might be a promising indicator for quantifying the TME and predicting the response to ICB immunotherapy in colon cancer.

## Methods

### Patients and Tissue Samples

144 pairs of cancer and adjacent normal tissues in total were included in this study. Tissue samples were obtained from patients with primary colon cancer who had undergone surgical resection at Changhai Hospital. This study was approved by the Clinical Research Ethics Committee of Changhai Hospital. The diagnoses for all patients were confirmed histopathologically of surgically resected tumors. TNM stage was defined according to the AJCC TNM Classification and histopathological classification of the resected tumors was performed. All fresh tissue samples were fixed with formalin and paraffinized for subsequent study. This study was conducted in compliance with the Declaration of Helsinki.

### Tissue Microarray and Immunochemistry

The tissues were made into formalin-fixed paraffin-embedded tissue microarrays and slides, after which we performed dewaxing using xylene and hydration using a grade alcohol series. After citric acid solution or EDTA buffer (pH 9.0) were used for antigen retrieval, 3% H_2_O_2_ was applied for the inhibition of endogenous peroxidase. Then the sections were incubated with 5% BSA for 30 min. Next, HRP-conjugated goat anti-rabbit IgG (1:200, Servicebio) was incubated as the secondary antibody for 50 min. Sections were then stained with 3,3-diaminobenzidine (DAB) (Dako). The primary antibodies used were listed as follows: The anti-biglycan primary antibody (diluted 1:2000, Abcam), anti-CD163 primary antibody (diluted 1:500, Servicebio), anti-FOXP3 primary antibody (diluted 1:500, Abcam). Protein expression was assessed with the value of the mean optical density (MOD) using Image-Pro Plus 6.0 software. The definition of BGN positivity was that the ratio of the value of MOD in the tumor tissue to that in the tumor-adjacent tissue of the same patient was greater than 1. Conversely, if the ratio was less than or equal to 1, it was considered BGN negative.

### Acquisition of Gene Expression Profiles

We downloaded the mRNA expression profiles of 435 colon adenocarcinoma (COAD) cases, 41 normal sample cases and the corresponding clinical data from TCGA (https://portal.gdc.cancer.gov/). The microarray datasets GSE4107, GSE110224, GSE4183 and GSE17536 were collected from GEO (Gene Expression Omnibus, http://www.ncbi.nlm.nih.gov/geo/). DEGs with upregulated or downregulated expression levels in GEO microarrays were analyzed with |log fold change (FC)| ≥ 1.5, and adjusted p-value < 0.05 as threshold. Gene expression data in these datasets were converted to transcripts per million (TPM) and processed for log (x + 1) normalization.

### Human Protein-Protein Interaction Analysis

The STRING database (http://string-db.org) was utilized to construct PPI networks of coexpressed genes with interaction scores > 0.4. For visualization, CytoHubba, a plugin from the open-source platform Cytoscape (version 3.8.2) (http://www.cytoscape.org/), was employed to analyze and calculate the network structure and weighted reconnections between potential hub gene nodes by built-in algorithms. Darker nodes represent higher scores, and proteins with high scores are more likely to be key proteins.

### Estimation and Bioinformatic Analysis of TIICs

The proportion of stromal-immune components in the TME of each sample was calculated with the ESTIMATE packages in R (29). The status of immune cell infiltration was acquired with the quanTIseq package in R, which performs an absolute quantification of 10 immune cell types from gene expression profiles (30). The results obtained were then validated by analysis with the ‘CIBERSORT’ R package (http://cibersort.stanford.edu/) (31), which has a preset LM22 leukocyte gene signature matrix to distinguish the proportion of immune cells. For in-depth exploration of the association between BGN expression and potential biological functions and pathways, we conducted GSVA analysis *via* the R package GSVA (32). In total, 14 immunity-related gene sets, which covered different processes of innate and adaptive response, were derived from the Molecular Signatures Database (MSigDB).

### Quantification of the Relative Abundance of TIICs and Prediction of the Response to Immunotherapy

The Immune Cell Abundance Identifier (ImmuCellAI) (33) (http://bioinfo.life.hust.edu.cn/ImmuCellAI#!/analysis) represents a new algorithm capable of estimating of the abundance of 24 TIICs and predicting the response of patients to immune checkpoint blockers therapy based on gene expression datasets. Colon cancer samples in TCGA were calculated with the GSVA analysis using the T-cell inflammatory (TIS) signature, which has been reported to be a genetic profile that effectively predicts the clinical response to pembrolizumab in various tumor types (34). Tumor immune dysfunction and exclusion (TIDE) (http://tide.dfci.harvard.edu/login/) (35), a computational algorithms that integrates data to assess mechanisms of two tumor immune escape. Patients with higher TIDE scores are more likely to experience from antitumor immune escape and consequently exhibit lower response rates to immune checkpoint blockers. In this study, TIS, ImmuCellAI and TIDE were performed for predicting the efficacy of ICBs in patients with colon cancer.

### Survival Analysis

The R package “survival and survminer” was used for the survival analysis We plotted survival curves by the Kaplan-Meier method, and utilized log rank test generated p-values with p < 0.05 considered significant.

### GO and KEGG Enrichment Analysis

DEGs was obtained by limma package (version: 3.40.2) of R software. GO and KEGG analyses were conducted by use of the ClusterProfiler package (version: 3.18.0) in R. Only terms with both p-values<0.05 and q-values<0.05 were considered significantly enriched.

### Gene Set Enrichment Analysis

Hallmark and C7 gene set v7.2 collections downloaded from the Molecular Signatures Database (MsigDB) were selected as the target sets, with which GSEA performed *via* gsea-4.1.0 software. The pathways were considered statistically enriched with p-values < 0.05 and false discovery rate (FDR q-value < 0.05).

### Statistical Analyses

Statistical analyses and figure generation were all executed in R (version 3.6.3), GraphPad Prism 8.0 and SPSS 25. Pearson’s correlation analysis was used to gauge the degree of correlation between variables. For all statistical analyses, p-values < 0.05 was considered statistically significant.

## Discussion

Given the safety and efficacy of tumor immunotherapy, A variety of ICBs have been approved by the FDA for the treatment of colorectal cancer. However, the process of mobilizing autoimmunity to achieve tumor eradication is delicate and complex, involving antigen presentation, T cell activation, tumor targeting, overcoming local suppression and tumor killing (36). The completion of these key steps determines the efficacy of immunotherapy. Thus, identifying new biomarkers that accurately indicate immune status and patient prognosis is of great value to make more robust therapeutic decisions in colorectal cancer.

Previous study has identified BGN as one of CNV-mRNA-protein correlated molecules in colorectal cancer patients with liver metastasis by integrative analysis of multi-omics data (37). In our study, three GEO datasets that included colon cancer tissue and normal tissue were examined for identifying differentially expressed genes. The PPI network subsequently showed that BGN was at the core of the filtered differential genes. Furthermore, in agreement with previously published findings (37, 38), we discovered the average mRNA expression of BGN was higher in colon cancer tissues comparing with normal tissues in TCGA as well as in our own sample set. In addition, our results reaffirm that BGN could serve as a potential informative molecule in the prognosis of colon cancer. Notably, BGN expression levels were also observed to be strongly correlated with tumor size and serosa invasion in our sample set. Interestingly, although prognostic information was not available for these patients, days of hospitalization for surgical treatment of colon cancer was calculated, as this might be a potential metric for clinical benefit that has been used in other studies (39, 40). Correlation analysis of the length of hospitalization suggested that patients with higher BGN expression experienced a correspondingly longer hospitalization stay. The above results again highlight the potential value of BGN and disease prognostic biomarkers.

The TME, which comprises multiple cell types and extracellular components, plays an indispensable role in tumorigenesis and progression and triggers immune escape (36, 41). Previous studies demonstrated that both immune and stromal infiltration were associated with prognosis in cancers (11, 26). Our findings revealed that higher immune infiltration was associated with worse clinical outcomes in colon cancer. Furthermore, BGN expression levels were found positively related to ESTIMATE, stromal and immune scores. As a pivotal component of the extracellular matrix, laboratory data provide evidence that BGN acts as a crucial role in driving chronic inflammation and promoting proliferation, migration, and angiogenesis in tumor progression (23, 42, 43). Our findings indicate that BGN-based outcome prediction in colon cancer may be related to the stroma and immune cells of the TME.

TAM infiltration contributes significantly to the immunosuppressive tumor microenvironment (44, 45). TAMs are the predominant TIICs and are commonly polarized into an immunosuppressive and tumor-promoting M2-like phenotype (46, 47). A recent study showed that M2 macrophages were the only cell type that notably predicted the prognosis of colon cancer with a hazard ratio (HR) > 100 (48). Thus, in tumors with large macrophage infiltrates, altering immune-suppressive characteristics into immune-promoting properties represents a promising approach for colon cancer treatment, as various strategies including targeting the anti-inflammatory cytokine IL-10, targeting pathogen recognition receptors and exogenous Beta-1,6 glucan supplementation have been reported in previous studies (49–51). The present investigation suggests that BGN might act as a potentially essential modulator of macrophages in colon cancer, which is evidenced by quanTIseq and CIBERSORT analysis of the proportion of TIICs. Moreover, BGN expression was found to be positively associated with the typical markers of M2 macrophages, but not significantly with M1 markers. Accordingly, BGN may be involved in initiation and maintenance of M2 polarization in macrophages of colon cancer. Translationally, targeting BGN represents a possible strategy to reorient TAMs toward the antitumor M1 phenotype. Treg cells are another vital group of immunosuppressive cells that mediate immune tolerance, inhibition of Tregs by targeting pivotal molecules could reverse tumor immunosuppression (52–54). In our present study, BGN-mediated immunosuppression may also involve Treg cells, which are functionally categorized into nTregs and iTregs (55). nTreg cells can interfere with cancer progression by reducing inflammation, while iTreg cells are the main suppressor cell subpopulation present at the tumor site that are responsible for suppressing the antitumor immune response (56). Interestingly, in our study, we found a contrasting correlation between these two subpopulations of Treg cells and BGN expression in colon cancer, with iTregs showing a positive trend distribution with BGN expression. These results illustrate a possible immunosuppressive role of BGN in the TME of colon cancer.

To obtain further insight into the underlying mechanisms and signaling pathways, DEGs between the high and low BGN expression groups were analyzed. As a result of GO and KEGG analyses, DEGs were concentrated in immune-related functions. Furthermore, GSEA and GSVA analyses showed that various immune response-suppressive gene sets and pathways were enriched in the BGN high expression group. Interestingly, a prior study has shown that BGN can significantly promote the expression of MHC-I molecules, enhance immunogenicity and thus promote the immune response in tumors (57). Conversely, in our study, high expression of BGN was proved to be implicated in the repression of both innate and adaptive immune responses in colon cancer. In particular, patients with high BGN expression similarly shared higher expression of immune checkpoint genes, since these molecules act as major negative regulators that inhibit T-cell proliferation and maintain T cells in an inactivated status (58, 59).

Currently, experiences from clinical studies and practices have demonstrated the clinical benefits of ICBs in a subset of patients with colon cancer, yet a substantial proportion of patients still rarely benefit from immunotherapy or relapse after a short-term benefit. The variation on the number, type and function of different immune cells in the TME might be critical factors in regulating the response to ICBs. It is thus hypothesized that the immune status of the TME, known as “cold” immunodesert tumors and “hot” inflammatory immunoinfiltrative tumors, correlates with the response of immunotherapy in cancer patients (34, 60). Consistent with this concept, high immune cell infiltration and high expression of immune checkpoints presented in BGN high expression subgroup suggested its potential clinical implication for predicting immunotherapeutic responsiveness. Moreover, patients in the high BGN subgroup attained higher TIS scores, which was reported to be related to the response to pembrolizumab. The response rate to immunotherapy in colon cancer patients was predicted by the ImmuCellAI and TIDE algorithms, both of which revealed that patients with high expression levels of BGN expression tended to be more likely to respond to immunotherapy.

Despite these promising findings, limitations still remain in this work. First, sample data were obtained from public sources as a result of lacking adequate clinical information to verify the prognostic impact of BGN and its predictive value on immunotherapy response in patients with colon cancer. Second, correlation analysis merely provides preliminary information of a relationship without identifying a causality between BGN and TIICs. BGN may display versatile and complex roles in the tumor immune response, which may depend on the cellular context according to previous reports and our study. Hence, *in vitro* and *in vivo* experiments will be needed to further elucidate the precise underlying molecular mechanisms of BGN in the tumor immune response.

In summary, this study provides preliminary evidence that BGN could serve as a valid biomarker for diagnosis, prognosis, and immunotherapy response prediction in patients with colon cancer. As each human tumor creates its own unique microenvironment, assessment of BGN expression represents a promising approach for identifying patients with the greatest potential to benefit from immunotherapy and a new venture into personalized therapy for cancer patients.

## Data Availability Statement

The datasets presented in this study can be found in online repositories. The names of the repository/repositories and accession number(s) can be found in the article/**Supplementary Material**.

## Ethics Statement

The studies involving human participants were reviewed and approved by the Clinical Research Ethics Committee of Changhai Hospital. The patients/participants provided their written informed consent to participate in this study.

## Author Contributions

ZH, SZ and XF conceived and designed the study. JE, HF, YS, JW, PP, LG, TX and YL collected and analyzed the data. ZH, SZ and JE wrote the original draft. ZH and HF revised the manuscript. YB, SW and ZL reviewed and edited the manuscript. All authors contributed to the article and approved the submitted version. All authors contributed to the article and approved the submitted version.

## Funding

YB is supported by the National Natural Science Foundation of China (grant 81873546 and 82170567), “Shu Guang” project of Shanghai Municipal Education Commission and Shanghai Education Development Foundation (grant 19SG30), the National Key R&D Program of China (grant 2018YFC1313103) and 234 Discipline Climbing Plan of Changhai Hospital, Second Military Medical University/Naval Medical University (grant 2019YXK004). S-LW is supported by the National Natural Science Foundation of China (no. 82100587), the Shanghai Sailing Program (grant 21YF1458700) and the Youth Star-tup Fund of Changhai Hospital, Second Military Medical University/Naval Medical University (grant 2019QNB02). TX is supported by grants from the National Natural Science Foundation of China (grant 81702373). XF is supported by grants from the National Natural Science Foundation of China (grant 81800479).

## Conflict of Interest

The authors declare that the research was conducted in the absence of any commercial or financial relationships that could be construed as a potential conflict of interest.

## Publisher’s Note

All claims expressed in this article are solely those of the authors and do not necessarily represent those of their affiliated organizations, or those of the publisher, the editors and the reviewers. Any product that may be evaluated in this article, or claim that may be made by its manufacturer, is not guaranteed or endorsed by the publisher.
