# Supplementary material for: Prognostic and Predictive Value of BGN in Colon Cancer Outcomes and Response to Immunotherapy
Source: Front Oncol. 2022 Jan 11;11:761030. doi: 10.3389/fonc.2021.761030 (PMC8790701; doi:10.3389/fonc.2021.761030)
Supplement: Supplementary file 1 [file DataSheet_1.docx]

**Supplementary Figure 1:** **The correlation between BGN expression levels and tumor microenvironment scores in different stages of colon cancer.**


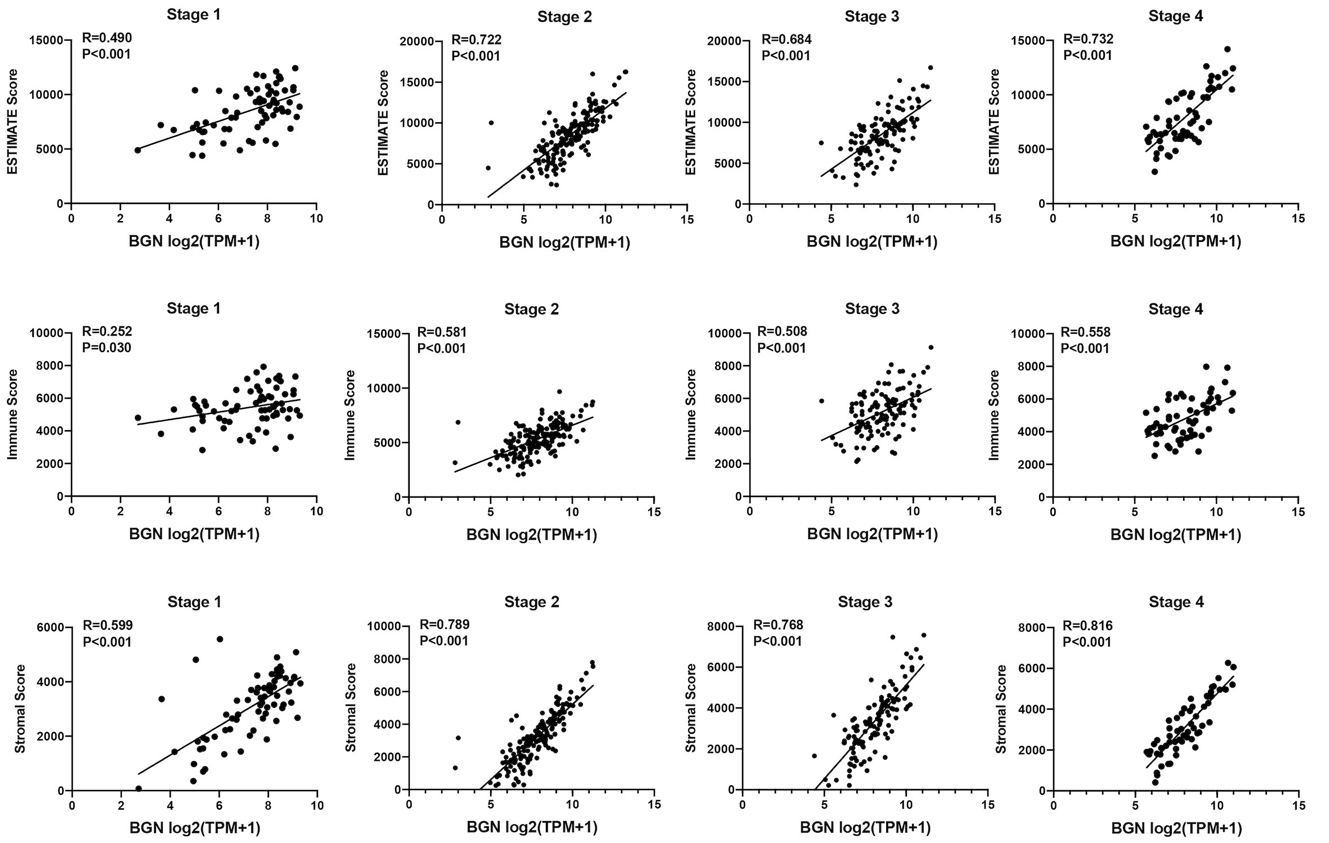


The p value was calculated by the Pearson correlation analysis. p < 0.05 was considered statistically significant.
