# Supplementary material for: Prognostic and Predictive Value of BGN in Colon Cancer Outcomes and Response to Immunotherapy
Source: Front Oncol. 2022 Jan 11;11:761030. doi: 10.3389/fonc.2021.761030 (PMC8790701; doi:10.3389/fonc.2021.761030)
Supplement: Supplementary file 5 [file Table_4.docx]

Supplementary Table 4. Enriched gene sets analysis.

| MSigDB collection | Gene set name | NES | p val | q val |
| --- | --- | --- | --- | --- |
| h.all.v6.2.symbols.gmt |  |  |  |  |
| BGN high expression | HALLMARK_ALLOGRAFT_REJECTION | 1.6387 | 0.0010 | 0.0014 |
|  | HALLMARK_COMPLEMENT | 1.5729 | 0.0010 | 0.0014 |
|  | HALLMARK_INFLAMMATORY_RESPONSE | 1.6433 | 0.0010 | 0.0014 |
|  | HALLMARK_INTERFERON_ALPHA_RESPONSE | 1.5366 | 0.0010 | 0.0014 |
|  | HALLMARK_INTERFERON_GAMMA_RESPONSE | 1.6421 | 0.0010 | 0.0014 |
|  |  |  |  |  |
| c7.all.v6.2.symbols.gmt |  |  |  |  |
| BGN high expression | GSE3039_CD4_TCELL_VS_ALPHABETA_CD8_TCELL_UP | 1.4213 | 0.0010 | 0.0027 |
|  | GSE9650_EXHAUSTED_VS_MEMORY_CD8_TCELL_UP | 1.3354 | 0.0010 | 0.0027 |
|  | GSE5099_CLASSICAL_M1_VS_ALTERNATIVE_M2_MACROPHAGE_DN | 1.2501 | 0.0090 | 0.0179 |
|  | GSE19512_NAUTRAL_VS_INDUCED_TREG_DN | 1.1790 | 0.0320 | 0.0457 |
|  | GSE2197_IMMUNOSUPPRESSIVE_DNA_VS_UNTREATED_IN_DC_UP | 1.2021 | 0.0190 | 0.0311 |

NES: normalized enrichment score; Gene sets with p-value <0.05 and FDR q-value <0.25 were considered as statistical significance. Only several leading sets enriched in BGN high expression both in HALLMARK and C7 were listed here due to the large number of enriched gene sets.
